# Spinodal Decomposition of Filled Polymer Blends: The Role of the Osmotic Effect of Fillers

**DOI:** 10.3390/polym16010038

**Published:** 2023-12-21

**Authors:** A. I. Chervanyov

**Affiliations:** Institute of Theoretical Physics, University of Münster, 48149 Münster, Germany; chervany@uni-muenster.de

**Keywords:** polymer blend, filler, spinodal

## Abstract

The reported work addresses the effect of fillers on the thermodynamic stability and miscibility of compressible polymer blends. We calculate the spinodal transition temperature of a filled polymer blend as a function of the interaction energies between the blend species, as well as the blend composition, filler size, and filler volume fraction. The calculation method relies on the developed thermodynamic theory of filled compressible polymer blends. This theory makes it possible to obtain the excess pressure and chemical potential caused by the presence of fillers. As a main result of the reported work, we demonstrate that the presence of neutral (non-adsorbing) fillers can be used to enhance the stability of a polymer blend that shows low critical solution temperature (LCST) behavior. The obtained results highlight the importance of the osmotic effect of fillers on the miscibility of polymer blends. The demonstrated good agreement with the experiment proves that this effect alone can explain the observed filler-induced change in the LCST.

## 1. Introduction

A relatively small fraction of solid fillers is experimentally known [1,2,3,4,5,6,7,8,9,10,11] to have significant effect on the phase separation and miscibility of polymer blends. Nano-fillers are often used to improve a variety of industrially important functional properties of polymer blends, such as electrical and thermal conductivity [12], piezoresistivity [13], electromagnetic interference shielding [14], and sensor ability [15] to name a few. Owing to their small translational entropy, the high-molecular-weight components of polymer blends are known [10,16] to be hardly miscible. It is well known that solid fillers can serve as compatibilizers [1,3,11] or, on the contrary, deteriorate [17] the miscibility of polymer blends. The presence of even a small amount of fillers can essentially affect the stability of the polymer blend composites used in the above applications. It is therefore imperative to understand which specific entropic and enthalpic factors, arising from the presence of fillers, can promote or suppress the miscibility and stability of polymer blends. A quantitative understanding of the above factors not only opens a route toward managing the phase separation and miscibility of polymer blends, but it also enables a control of the morphology [18] of phase domains. Gaining control over the morphology of multi-component polymer systems is important [19], in particular, for the production of stable hybrid polymer-based materials.

Despite great practical interest, the theoretical studies of the effect of fillers on the stability, phase separation, and miscibility of polymer blends are rather scarce. In his early work on the subject, Warren [20] addressed the effect of the polydispersity of polymers on the phase separation in colloid-polymer mixtures. Conceptually, that study is a generalization of a previous work [21] on the phase behavior of monodisperse polymer-colloid mixtures, which built upon the ideas of the free volume theory. By construction, that theory operates with the free volume left by dense colloidal system for polymers coils, where these coils can move freely. Each polymer coil is considered to be a separate unit of a spherical shape that has a fixed radius. In addition, the coils are assumed to be ideal (non-interacting). The above assumptions make Warren’s theory more appropriate in the case of a dilute solution of polymers in dense colloidal systems. In the present work, we consider just the opposite case of dilute concentrations of solid fillers in dense polymer blends whose constituents interact with each other. In addition, our model covers the excluded volume interaction of a dense polymer blend with fillers described below.

In a celebrated series of works (see [10,17,22] and references therein), Lipatov, Nesterov, et al. used the Flory–Huggins theory to quantitatively rationalize the effect of fillers on the phase separation of polymer blends. In their rather simplistic approach, the fillers are treated as a continuum solvent phase interacting with the blend components through the respective Flory–Huggins parameters. In the above work, the translational entropy of polymer chains is considered to be negligible relative to the enthalpic interactions that therefore ultimately dominate the phase separation process. The main advantage of the Lipatov–Nesterov (LN) model is its simplicity. Although being very useful for the qualitative evaluation of a phase diagram of polymer–polymer–particle systems, this approach still overlooks many of the critical features important for the relevant effects. In particular, the shape and size of fillers, as well as the characteristics of polymers (e.g., polymerization degree and monomer size), fall out of the scope of this approach. This omission does not make possible to describe, in particular, the osmotic (excluded volume) and surface effects of fillers on the stability of polymer blends. As is shown in the present work, the above osmotic effect is critical for understanding the influence of fillers on the phase separation of filled polymer blends.

A step forward, relative to the described pioneering LN model, was undertaken by Ginzburg [23] and He, Ginzburg, and Balazs [24], who extended theirs and others’ [25,26] approach for filled diblock copolymer systems over the case of polymer blends. The main advantage of this simple theory is that it takes into account the finite radius *R* of a filler by introducing surface interactions between fillers and polymers. The respective term in the interactions’ free energy ∼$R^{2}$ is postulated based on the plausible arguments relevant to a diblock copolymer system rather than a polymer blend. In particular, the entropic part of the surface interactions are deduced from stretching free energy [25,27], which implies a brush-like structure of the stretched diblock copolymer chains in the strong segregation limit. In addition, this single surface interaction term does not include the osmotic effect ∼$R^{3}$ caused by the volume excluded by a filler to polymers. As we demonstrate in the present work, this term plays a key role in the effect of fillers on the miscibility of polymer blends. Finally, the described model is restricted to incompressible polymer blends. As the finite compressibility of a blend is known [28,29,30] to be a prerequisite for the lower critical solution temperature (LCST) behavior [28,29,30,31] of this blend, this model is not applicable to the LCST phase transitions considered in the present work.

In the present work, we develop a model that describes the effect of fillers on the stability, miscibility, and phase separation of a polymer blend, which is based on rigorously taking into account the abovementioned osmotic effect of fillers on the thermodynamic state of this blend. The main advantages of this model are its wider applicability and the rigorous description of the effect of fillers on the thermodynamics of a polymer blend. A wider applicability of our model relies on taking into account the equation of state effects [32], such as the finite compressibility of a blend. This feature makes it possible, in particular, to adequately describe the effect of fillers on the LCST behavior of blends that is often observed in experiments [3,4,6,7,8,9,33,34]. The mentioned rigorous description of the thermodynamic effects caused by the presence of fillers relies on the consistent calculation of the excess thermodynamic functions [35,36]. This calculation makes it possible, in particular, to consistently calculate the main, osmotic contribution to the effect of fillers on the stability of polymer blends that was omitted in the previous work.

For the sake of transparency, in the present work, we restrict ourselves to considering only the osmotic part of the polymer–filler interactions that arise in cases where a filler is neutral to both of the polymer species comprising a blend. In addition, we consider only the dilute concentrations of fillers in a blend, which greatly simplifies the associated mathematical development. It is important to note that the above limitations, imposed only for transparency and simplicity in the technical development, do not belong to the intrinsic restrictions of the developed model. More technically demanding cases covering the enthalpic (e.g., adsorption) interactions between fillers and polymers, as well as those dealing with large concentration of fillers, will be reported elsewhere.

The paper is organized as follows. Section 2 summarizes the technical details of the developed theoretical model and is subdivided into three subsections. Section 2.1 derives the spinodal condition for a filled polymer blend. Section 2.2 calculates the excess thermodynamic quantities of a blend, caused by the presence of fillers. Section 2.3 analyzes the effect of fillers on the phase separation of a polymer blend. Section 3 discusses the obtained findings and describes the comparison with the experiment. A short summary of the performed studies is given in Section 4.

## 2. Theory

### 2.1. The Condition of the Spinodal Decomposition of Filled Polymer Blends in Infinite Dilution Approximation

We consider a polymer blend of two homopolymers, which have polymerization degrees $r_{1}$ and $r_{2}$, filled with spherical fillers that have a volume $v_{R}$. The volume fraction φ of fillers is assumed to be much smaller than that of its polymer counterparts in order to best mimic typical experimental conditions [3,10]. The considered small φ justifies applying the infinite dilution approximation with respect to fillers, which greatly simplifies theoretical development. Under this approximation, both the direct and effective (e.g., polymer-mediated [37]) interactions between fillers can be neglected. The role of fillers for the stability of a blend, therefore, reduces to the purely osmotic effect described in the next subsection in detail. As has been shown in our previous work in [38,39,40], this osmotic effect is responsible for the localization of fillers at the interfaces between the microphases of diblock copolymer systems. We surmise that a similar effect explains the interfacial localization of fillers in the polymer blends observed, e.g., in [3] for the (PS)-grafted silica nanofillers in the polystyrene-co-acrylonitrile (SAN)- poly(methil matacrylate) (PMMA) blend. The adopted theoretical assumptions, therefore, mimic the described conditions of the experiment that we use for the comparison with our theoretical results in Section 3.

The generic conditions of the stability of a three-component system against diffusion are well known [41]:

(1) $$\mu_{11}\geq 0,\;\mu_{33}\geq 0,\;\mu_{11}\mu_{33}-\mu_{13}^{2}\geq 0,$$

where $\mu_{ij}=\partial_{M_{j}}\mu_{i}\left(M_{i},P,T\right)$ are the partial derivatives of the chemical potentials $\mu_{i}$ of the blend species with respect to the number of molecules $M_{j}$ at given pressure *P* and temperature *T*. For further development, it is instructive to apply the above stability conditions to component 1 of a polymer blend and filler particles that are referenced by indexes 1 and 3 in Equation (1), respectively.

Note that, in the absence of filler particles, the above stability condition reduces to $\mu_{11}\geq 0$. This inequality can be conveniently recast in terms of a reduced blend density η=Mrv/V, and the monomer 1 mole fraction $\phi_{1}\equiv \phi =1-\phi_{2}=M_{1}r_{1}/Mr$ to be written as

(2) $$\partial_{\phi }\left(r_{1}^{-1}\mu_{1}-r_{2}^{-1}\mu_{2}\right)-T\eta^{-1}{\tilde{\kappa }}_{T}{\left(\partial_{\phi }\tilde{P}\right)}^{2}\geq 0,$$

where *V* is the volume occupied by a blend at pressure *P*; $Mr=M_{1}r_{1}+M_{2}r_{2}$ is the total number of the polymer segments; $v_{i}\equiv v$ is the hard-core (h.c.) volume of the monomer of species *i* that is assumed to take the same value *v* for all polymer species; and $\tilde{P}=vP/T$ and ${\tilde{\kappa }}_{T}=-{\left(V\partial_{V}\tilde{P}\right)}^{-1}$ are the reduced pressure and isothermal compressibility, respectively. The first, main term in the left hand side (l.h.s.) of the inequality given by Equation (2) originates from the translational entropy of the polymers and the enthalpic interactions between them. The second term is the correction due to the finite compressibility of a blend. The derivation of Equation (2) is given in Appendix A. Note that this equation gives the genetic stability condition for binary mixtures that is valid for any model for pressure *P* and chemical potentials $\mu_{i}$ (i=1,2). For the Sanchez–Lacombe model for these quantities, which are described below in detail, this condition is reduced to that given by Equation (57) in [32] in the considered case $v_{1}=v_{2}$.

In the considered infinite dilution approximation, the stability conditions given by Equation (1) does not reduce to its filler-free counterpart, which is given by Equation (2). Still, this latter condition with pressure *P* and the chemical potentials $\mu_{i}$ (i=1,2), calculated by taking into account the presence of fillers, gives a leading order contribution to the stability condition of a filled polymer blend. It is, therefore, instructive to analyze this condition in detail.

To rationalize the spinodal condition in Equation (2) for a pure (unfilled) polymer blend, we use the celebrated Sanchez–Lacombe theoretical prediction for the required thermodynamic quantities given by [28,32]

(3) $${\tilde{P}}_{0}=\eta \left(p+\eta Q\left(\eta \right)\right)-T^{-1}\eta^{2}\epsilon \left(\phi \right),$$

(4) $$\mu_{i}^{0}=T\left(\log\left(\eta \phi_{i}\right)+1-pr_{i}+r_{i}\left(\eta^{-1}-1\right)\log\left(1-\eta \right)\right)+r_{i}\eta \left(T\eta^{-2}{\tilde{P}}_{0}+{\left(1-\phi_{i}\right)}^{2}\Delta \epsilon -\epsilon_{ii}\right)$$

for pressure $P_{0}$ of a pure polymer blend and chemical potential $\mu_{i}^{0}$ (i=1,2) of the polymer molecules of species *i* of this blend, respectively. Hereafter, the Boltzmann constant *k* is adsorbed into temperature so that kT≡T, ${\tilde{P}}_{0}=vP_{0}/T$ is the reduced pressure, $\Delta \epsilon =\epsilon_{11}+\epsilon_{22}-2\epsilon_{12}$, $p=\sum_{l=1,2}\phi_{l}r_{l}^{-1}$, $Q=-\left(\eta +\log\left(1-\eta \right)\right)/\eta^{2}$, $\epsilon \left(\phi \right)=\sum_{m,l=1,2}\epsilon_{ml}\phi_{m}\phi_{l}$ ($\phi_{2}\equiv 1-\phi_{1}$) is the total polymer interaction energy, and $\epsilon_{ml}$ is the interaction energies between the polymer species *m* and *l* (m,l=1,2). Note that the reduced pressure ${\tilde{P}}_{0}$ given by Equation (3) is directly related to the reduced quantities ${\tilde{P}}_{SL}$ and ${\tilde{T}}_{SL}$, which are defined by Equation (37) in [32] by ${\tilde{P}}_{0}={\tilde{P}}_{SL}/{\tilde{T}}_{SL}$ (subscript SL is added to mark the quantities defined in [32]).

Substituting the expressions given by Equations (3) and (4) into the l.h.s. of the inequality (2) and equating the result to zero gives the equation for the spinodal of a pure polymer blend. Note that this equation is not closed as it contains three independent variables η, ϕ, and *T*. One of these variables (e.g., η) must be excluded by making use of the equation of state given by Equation (3). An additional, commonly used [28,29,30] approximation is to consider only the zero-pressure isobar that approximates the atmospheric pressure conditions that are typically used in experiments. Under these conditions, the l.h.s of Equation (3) is negligible relative to its right hand side (r.h.s.), which leads to a simple relation among η, ϕ, and *T* of the form

(5) $$\epsilon \left(\phi \right)=T(p\eta^{-1}+Q\left(\eta \right)).$$

This relation can be readily used to close the spinodal condition for a pure polymer blend. The thus obtained spinodal equation is analyzed in Section 3.1 in detail.

The presence of fillers causes the correction to the spinodal condition given by Equation (2). This correction is to be deduced from the full stability condition given by a set of inequalities in Equation (1). In the adopted infinite dilution approximation, this full stability condition can be significantly simplified. As a first step of this simplification, we note that the second inequality in Equation (1) is always fulfilled, as the largest, ideal component $\partial_{M_{3}}\log M_{3}∼1/M_{3}$ of $\mu_{33}$ is always positive. The first inequality in Equation (1) is, therefore, always fulfilled when the third inequality is fulfilled. The only non-trivial stability condition to be analyzed is thus given by the third inequality in Equation (1). Further simplification of this inequality requires knowledge of the excess thermodynamic quantities caused by the presence of fillers, e.g., the excess chemical potentials $\Delta \mu_{i}$ (i=1,2) of the polymer species, and the excess pressure ΔP of a blend. The calculation of these quantities is performed in the next section.

### 2.2. Osmotic Effect of Fillers on the Thermodynamics of a Polymer Blend

In order to express the spinodal condition given by the third inequality in Equation (1) in the explicit form, one needs to calculate the corrections to the pressure and chemical potentials of a polymer blend that are caused by the presence of fillers. It is important to note that these corrections must be calculated under the isothermal-isobaric conditions that correspond to the process of mixing.

In the calculation of the above-described excess thermodynamic quantities, we restrict ourselves to the simplest case where the presence of fillers causes only the osmotic effect on the thermodynamic state of a blend. As is shown in what follows, even this basic effect causes quite non-trivial corrections to the thermodynamic quantities and spinodal condition of this blend. In the absence of any polymer–filler and filler–filler interactions, the role of fillers reduces to creating an additional osmotic pressure ΔP in a polymer blend. In the considered infinite dilution limit, this pressure can be deduced directly from its pure blend counterpart, which is given by Equation (3). At a constant pressure, the presence of fillers changes the volume available to the blend by the value of the h.c. filler volume $M_{3}v_{R}$. Up to the leading order in the filler volume fraction, the reduced pressure $\tilde{P}\equiv Pv/T$ of a filled polymer blend is therefore given by

(6) $$\tilde{P}=v_{3}^{-1}\varphi \eta +{\tilde{P}}_{0}\left(\eta {\left(1-\varphi \eta \right)}^{-1},\phi \right)\approx {\tilde{P}}_{0}\left(\eta ,\phi \right)+\Delta \tilde{P},\;\Delta \tilde{P}=\varphi \eta (v_{3}^{-1}+{\tilde{\kappa }}^{-1}),$$

where $\varphi =M_{3}v_{R}/Mrv$ is the h.c. volume fraction of fillers, $v_{3}=v_{R}/v$ is their reduced volume, and $\tilde{\kappa }={\left(\eta \partial_{\eta }{\tilde{P}}_{0}\right)}^{-1}\equiv -{\left(V\partial_{V}{\tilde{P}}_{0}\right)}^{-1}$ is the reduced isothermal compressibility of a pure polymer blend. The first, ideal term $v_{3}^{-1}\varphi \eta$ in the r.h.s. of Equation (6) arises from the translational entropy of fillers.

The chemical potential of fillers can be straightforwardly obtained from Equation (6) by a standard procedure [41] of integrating $\tilde{P}$ over volume and by differentiating the result with respect to the number of fillers $M_{3}$. The result of this calculation reads

(7) $$\mu_{3}=T\log\left(\phi \eta \right)+v_{3}T{\tilde{P}}_{0}\left(\eta ,\phi \right).$$

The obtained expression for the chemical potential of fillers has a transparent physical meaning. The first term in the r.h.s. of this equation arises from the translational entropy of fillers. The second term describes the minimal work required to create a cavity of volume $v_{R}$ against the pressure $P_{0}$ exerted by a polymer blend.

The excess chemical potentials of the polymer blend components caused by the presence of fillers can be readily obtained by integrating the Maxwell relations $\partial_{M_{3}}\mu_{i}=\partial_{M_{i}}\mu_{3}$. In the infinite dilution limit with respect to fillers, one finds

(8) $$\mu_{1}=\mu_{1}^{0}+\varphi \Delta \mu_{1},\;\Delta \mu_{1}=r_{1}\eta T\left(\partial_{\eta }+\phi_{2}\eta^{-1}\partial_{\phi }\right){\tilde{P}}_{0}\left(\eta ,\phi \right),$$

where the chemical potential $\mu_{1}^{0}$ of component 1 of a pure polymer blend is given by Equation (4). When the pressure of a polymer blend is given by the simple expression in Equation (3), the excess chemical potential $\Delta \mu_{1}$ simplifies to

(9) $$\Delta \mu_{1}=\eta \left(T\left(1+r_{1}\eta {\left(1-\eta \right)}^{-1}\right)-2r_{1}\eta \sum_{i=1,2}\epsilon_{1i}\phi_{i}\right).$$

The expressions for the excess thermodynamic quantities caused by the presence of fillers given by Equations (6)–(9) are to be used in Equation (1) to obtain the spinodal condition for a filled polymer blend.

### 2.3. The Effect of Fillers on the Spinodal Decomposition of Polymer Blends

As a final step of the present theoretical development, the obtained expression for the excess chemical potentials given by Equations (7)–(9) are used to determine the explicit form of the spinodal condition given by Equation (1). Taking the derivatives of the chemical potentials with respect to number of molecules $M_{i}$ at a constant pressure results in quite cumbersome analytical expressions in the form of functions $\mu_{ij}\left(T,\eta ,\phi ,\varphi \right)$ (i,j=1,2). The above calculations are simplified by the symmetry relation $\mu_{13}=\mu_{31}$ (i,j=1,3) so that only the three derivatives $\mu_{11}$, $\mu_{33}$, and $\mu_{13}$ of the four are independent. Further, the obtained expressions for $\mu_{ij}$ are substituted into the l.h.s $\mu_{11}\mu_{33}-\mu_{13}^{2}$ of the spinodal condition given by the third inequality in Equation (1). The resulting expression is set equal to zero to obtain the spinodal equation of a filled polymer blend in the form S(T,η,ϕ,φ)=0. This equation determines the spinodal curve that delineates the regions of stability and instability of a polymer blend in the (ϕ,T)-plane for any given pressure *P* and filler fraction φ.

Since the resulting exact expression for *S* is enormously cumbersome, we restrict ourselves to showing the more tractable “long chain” limit $r_{i}\to \infty$ of the above expression. This reads as

(10) $$\begin{array}{c} S\equiv \left(2\delta \epsilon -T{\left(1-\eta \right)}^{-1}\Delta \epsilon \right)\left(1-v_{3}\varphi \eta^{3}\left({\left(1-\eta \right)}^{-1}-2T^{-1}\epsilon \right)\right)-\\ \varphi T\eta^{2}{\left(1-\eta \right)}^{-2}{\left(1-\phi \right)}^{-1}\left(\epsilon_{11}\left(1+\phi \right)-2\epsilon_{12}\phi -\epsilon_{22}\left(1-\phi \right)\right)=0, \end{array}$$

where $\delta \epsilon =\epsilon_{11}\epsilon_{22}-\epsilon_{12}^{2}$.

The effect of fillers on the spinodal of a polymer blend is described by the terms proportional to φ in Equation (10). Those of these terms that contain energetic parameters $\epsilon_{ij}$ describe the effect of the enthalpic interactions between the alike and unlike polymer species. The terms and multipliers proportional to ${\left(1-\eta \right)}^{-1}$ describe the effect of a finite compressibility of a polymer blend. Note that the above enthalpic- and compressibility-induced terms have different signs. The presence of fillers can, therefore, increase or decrease the spinodal temperature of a blend depending on the relation among the four parameters $\epsilon_{ij}$ (i,j=1,2), $v_{3}$ and the two variables ϕ and η. The sign of the effect of fillers on the stability and miscibility of a blend is to be investigated for each selected experimental setting. One example of such an investigation is given in Section 3.2.

Note that the present simplified expression for *S* is practically useful for evaluating the lower critical solution temperature (LCST) spinodal of a filled polymer blend. This LCST spinodal is determined by the balance between the described effects of the enthalpic interactions between the polymer species and finite compressibility of a polymer blend. Recall that it is this type of spinodal, often observed in experiments, that we aim to quantitatively describe in the present work. The effect of the translational entropy of polymers, which is neglected in the approximate Equation (10), gives rather a negligible contribution of the order of $1/r_{i}$∼0.001 under typical LCST experimental conditions. This effect, however, cannot be neglected when analyzing the upper critical solution temperature (UCST) spinodal of a filled polymer blend. It is, therefore, worth noting that the shown approximate spinodal equation has rather limited applicability in analyzing the UCST spinodal, which is known [32] to be mainly determined by the competition between the described polymer translational entropic and enthalpic effects.

It is also important to caution that care must be exercised when using the spinodal equation given by Equation (10), as the polymer cross-species interaction parameter $\epsilon_{12}$ is yet an undetermined function of temperature. This function is to be modeled or determined from the experimental data, as is described in Section 3.1.

To avoid the described limitations, in what follows we use the exact expression for S(T,η,ϕ,φ), obtained with the help of the software Mathematica 13.0. The spinodal equation based on the thus-obtained function S(T,η,ϕ,φ) is not yet sufficient to obtain the spinodal in the desirable form T(ϕ,φ). To close this equation, one needs to use the equation of state to calculate the zero-pressure isobar, as described in Section 2.1. This isobar is then used to express the reduced blend density η as a function of temperature *T* and volume fractions ϕ and φ. The required relations among η, *T*, ϕ, and φ, which are obtained from Equation (6), read as

(11) $$\eta \epsilon \left(1+2\eta \varphi \right)=v_{3}^{-1}\varphi T+pT\left(1+\eta \varphi \right)+\eta T\left(Q\left(\eta \right)+\eta \varphi {\left(1-\eta \right)}^{-1}\right),$$

where ϵ, *p*, and Q(η) are defined below Equation (4). Note that $\epsilon_{12}$ in ϵ is a function of temperature (see Section 3.1).

Simultaneous Equations (10) and (11) are to be solved numerically to determine the spinodal T(ϕ) for the selected set of five parameters $r_{i}$, $\epsilon_{ii}$ (i=1,2) and φ known from the experimental conditions. The sixth parameter, i.e., the cross-species interaction energy $\epsilon_{12}$, is determined by the fitting procedure described in Section 3.1.

## 3. Discussion and Comparison with the Experiment

### 3.1. Determination of the Cross-Species Interaction Parameter of a Pure Blend

The Sanchez–Lacombe expressions for the pressure and chemical potentials of the unfilled (pure) polymer blend given by Equations (3) and (4) contain the only undetermined parameter: the cross-species interaction energy $\epsilon_{12}$. The four remaining parameters, i.e., the interaction energies $\epsilon_{ii}$ between the alike polymer species and the polymerization degrees $r_{i}$ (i=1,2), can be derived from the properties of the pure components. One effective way to evaluate the above parameters is to fit the one-component analog of Equations (3), written for each blend component, to the respective experimental PVT-data [29,42]. Note that the thus-obtained interaction parameters $\epsilon_{ii}$ are given [28,29,30] in the form of the characteristic temperatures $T_{i}$. Care must be exercised when deriving $\epsilon_{ii}$ from $T_{i}$, as $\epsilon_{ii}$ are, in effect, the parameters of the SL lattice theory that contains a somewhat arbitrary scaling multiplier *z*: the coordination number of the used lattice. Note that $\epsilon_{ii}$ and $T_{i}$ are related through $z\epsilon_{ii}=kT_{i}$. Fortunately, the obtained results do not depend essentially on the chosen value of *z* for z≥4. We chose to work with z=12, which is specific to the face-centered cubic and hexagonal close-packed lattice structures.

A spinodal, calculated using the temperature- and monomer fraction-independent $\epsilon_{12}$, is known [28,29,30] to show only UCST behavior [29,30,31]. This spinodal fails to explain the experimental observations, where the opposite LCST behavior is observed (see, for example, [28,29]). To mimic the above experimental conditions, one therefore needs to model the dependence of $\epsilon_{12}$ on temperature. One popular model [28] relies on the evaluation of the temperature dependence of $\epsilon_{12}$ by taking into account the strong specific interactions between the unlike components of a polymer blend. Although the temperature dependence of $\epsilon_{12}$, imposed by the specific interactions, can cause the occurrence of the LCST-type spinodal, having these interactions is not a necessary condition for LCST behavior to emerge. In particular, the simple linear dependence $\epsilon_{12}\left(T\right)$ has also been shown to cause [29,30] the LCST behavior of polymer blends. The physics behind this behavior, therefore, cannot be unequivocally traced back to the specific interactions. As the physical reasons for the LCST behavior of polymer blends on the molecular level have not yet been determined, we take a more straightforward route to the determination of $\epsilon_{12}$ that does not rely on any physical concept. Specifically, we directly fit the calculated $\epsilon_{12}$-dependent spinodal curve to the experimental data, as described in what follows.

The mentioned fitting procedure has been performed by the numerical calculation described below. This calculation relies on the spinodal condition given by Equation (2) with the chemical potential $\mu_{i}$ and pressure *P* of an unfilled polymer blend, which are given by Equations (3) and (4), respectively. Recall that this spinodal condition relates the three variables, i.e., temperature *T*, blend reduced density η, and monomer-1 fraction ϕ, for any given set of the five parameters $T_{i}$, $T_{12}=\epsilon_{12}/k$, $r_{i}$ (i,j=1,2) described in the beginning of this section. $T_{12}$, the characteristic temperature of the cross-species interactions, is the key parameter that is to be determined by the proposed fitting procedure. Hereafter, indexes 1 and 2 correspond to polystyrene-co-acrylonitrile (SAN) and poly(methil matacrylate) (PMMA) polymer blend components, respectively. The values of the remaining parameters can be taken from the experimental data. Here, we use the values of the reduced temperatures $T_{1}=731$ K and $T_{2}=699$ K, which are given in [43]. The h.c. monomer volume $v=1.57\times 10^{-2}$ nm^3^ has been evaluated by applying the Berthelot rule (geometric mean) to the values of the h.c. mer volumes of the blend components given in [43]. The values $r_{1}=2658$ and $r_{2}=1592$ have been directly derived from the molecular weights of the polymer materials used in [3]. Further, variable η can be excluded by making use of the zero-pressure isobar given by Equation (5). As a result, one obtains the implicit spinodal equation S(T,ϕ)=0 for any given value of parameter $T_{12}$. By substituting the coordinates (ϕ,T) of the experimental transition temperature points, shown in Figure 6 in [3], into the above equation, one finds the values of $T_{12}$ for each such point.

**Figure 1:**
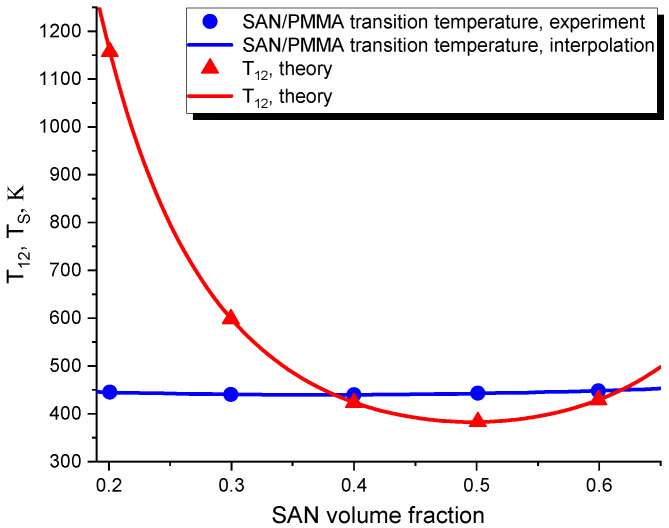
The characteristic temperature $T_{12}$ of the cross-species interactions, obtained by the fitting procedure described in the text. The experimental transition temperatures $T_{S}$ were obtained in [3], and their polynomial interpolation is shown for reference.

One example of using the described fitting procedure is illustrated in Figure 1, which shows the transition temperature points (marked by blue circles) that have been determined in the abovementioned experiment in [3]. As the shown experimental points are rather scarce, we have interpolated these points by a simple polynomial fit, which is shown as a separate blue curve in Figure 1. The obtained interpolated spinodal has been then fed to the described fitting procedure.

The scatter points marked by red triangles in Figure 1 show the values of $T_{12}$ that were obtained from the corresponding spinodal points. In addition to the above scatter of the $T_{12}$ points directly derived from the corresponding experimental transition temperature points, Figure 1 also shows the red curve $T_{12}\left(T\left(\phi \right)\right)$, where T(ϕ) is the spinodal function. This curve has been derived from the interpolated experimental points with the objective to give a more comprehensive prediction of $T_{12}$ for the whole range of ϕ. As can be elucidated from Figure 1, $T_{12}$ non-monotonically depends on ϕ along the spinodal T(ϕ). At smaller values of ϕ, $T_{12}$ has a value of the order of 1000 K, which is much larger than that of the corresponding spinodal temperature $T_{S}$. With increasing ϕ to 0.3, $T_{12}\left(\phi \right)$ steeply decreases until it reaches the value of the order of $T_{S}$. Upon further increasing ϕ, $T_{12}$ reaches its minimum of 383 K at ϕ≈0.5. In the region where ϕ>0.3, the value of $T_{12}$ is therefore found to be of the order of the corresponding spinodal temperature. Interestingly, $T_{12}$ has a minimum at ϕ∼0.5, which corresponds to the symmetric blend composition.

The obtained fitted values of $T_{12}$ ($\epsilon_{12}$) are to be used to investigate the spinodal of the SAN/PMMA polymer blend in the presence of the silica fillers described in the next section.

### 3.2. The Spinodal of a Filled Polymer Blend

The cross-species interaction parameter $\epsilon_{12}$, obtained in the preceding section, provides a critical input that is necessary for the calculation of the spinodal of a filled polymer blend. To perform this calculation, we rely on Equations (10) and (11), derived in Section 2.3. In a full analogy with the case of a pure polymer blend that is considered in Section 3.1, one needs to solve these simultaneous equations to deduce the spinodal equation of the form S(T,ϕ)=0. Equivalently, η is to be excluded by numerically solving Equation (11) that describes the zero-pressure isobar, and then substituting the result into Equation (10).

The above-described calculation has been performed using the values of parameters $\epsilon_{ii}$, $r_{i}$ (i=1,2), which are given in Section 3.1 for the pure polymer blend SAN/PMMA. Recall that the cross-species interaction parameter $\epsilon_{12}$ ($T_{12}$) has been obtained by fitting the theoretical spinodal to the experimental transition points measured in the system that is characterized by these parameter values. Since $\epsilon_{12}$ ($T_{12}$) depends on temperature only, the obtained values of $T_{12}\left(T\right)$ could be used for the polymer volume fractions ϕ that deviate from those observed in the experimental transition points.

The only two additional parameters needed for the present calculation of the spinodal of a filled polymer blend are filler volume $v_{R}$ and filler h.c. fraction φ, which are defined in Section 2.1. These parameters have been derived from the silica filler radius of 30nm and the filler weight fraction of 0.03, used in the experiment in [3].

**Figure 2:**
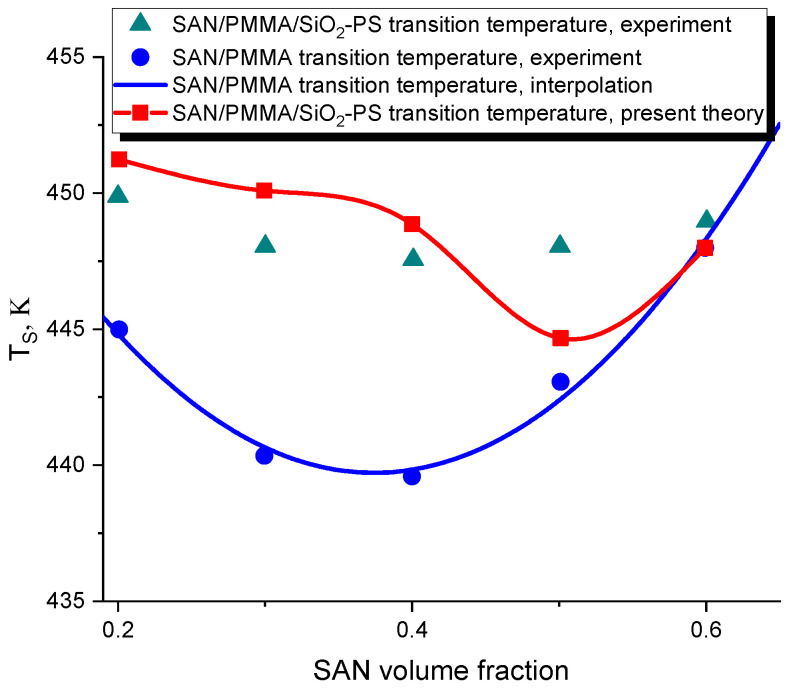
Spinodal of the SAN/PMMA blend filled with silica fillers: theory vs. experiment. See explanation in the text. The experimental rheological transition temperatures $T_{S}$ obtained in [3] and their polynomial interpolation for the unfilled SAN/PMMA blend are shown for reference.

The results of the performed calculation are illustrated in Figure 2. For the sake of referential convenience, Figure 2 shows the experimental transition temperature points of the pure SAN/PMMA blend and their polynomial interpolation, marked by the blue circles and the blue curve, respectively. The scatter points marked by the green triangles show the experimental transition points for the filled $SAN/PMMA/(SiO_{2}-PS)$ blend. It is important to mention that the $SiO_{2}$ fillers used in the experiment in [3] were grafted with relatively short PS chains. These fillers were found to be localized at the interfaces, thus showing no preference to the SAN- and PMMA-rich phases of the phase-separated blend. This case, therefore, corresponds with the assumptions of the developed theory that assumes that fillers have no enthalpic preference for any of polymer species.

The scatter points in Figure 2 marked by the red squares show the results of our calculation of the spinodal of the filled $SAN/PMMA/(SiO_{2}-PS)$ blend. In this calculation, we use the parameters of a pure SAN/PMMA blend, including the cross-interaction energy $\epsilon_{12}$. The main difficulty in the calculation arises from the fact that, at the same volume fraction ϕ, $\epsilon_{12}\left(T\right)$ has different values at the spinodal transition temperatures $T_{S}$ and $T_{S}^{0}$ of the filled and unfilled blends, respectively. $T_{S}$ must be self-consistently determined from Equations (10) and (11) by adjusting the values of $\epsilon_{12}$ for given ϕ and $T_{S}^{0}$.

In addition to using several well-separated experimental cloud points as an input for the described calculation, we performed the same calculation based on the polynomial interpolation of these points, as shown by the blue curve in Figure 2. The thus-obtained spinodal is shown by the red line.

Figure 2 demonstrates that the developed theory correctly predicts the sign of the effect of fillers on the transition temperature $T_{S}\left(\phi \right)$ that delineates the regions of stability and instability of a polymer blend. Specifically, the presence of fillers has been found to cause an increase in $T_{S}\left(\phi \right)$ in the whole range of ϕ, thus enhancing the stability of the blend. The significance of this effect essentially depends on the fractions of polymer species that are quantified by ϕ, as is reflected by the non-monotonic dependence $T_{S}\left(\phi \right)$.

In addition, the theoretical prediction gives an adequate quantitative estimate of the transition temperatures in the whole range of blend compositions ϕ without using any adjustable parameters. The theory predicts a slightly steeper, compared to the experimental observations, dependence of $T_{S}\left(\phi \right)$ that features a deeper minimum. As can be seen from Figure 2, in the region where ϕ<0.4, the theoretical prediction slightly overestimates the magnitude of the effect of fillers on the transition temperature that has been measured in the experiment. On the other hand, the theory correctly predicts the trend of changing this magnitude upon increasing the SAN volume fraction ϕ. As observed both theoretically and experimentally, increasing ϕ in the region where ϕ<0.4 results in a slight increase in the shift of the transition temperature $\Delta T_{S}$ caused by the fillers. In the region where 0.4<ϕ<0.6, on the contrary, increasing ϕ causes diminishing the effect of fillers on $\Delta T_{S}$. While the theory predicts that the magnitude of the effect of fillers steeply decreases for ϕ>0.4, the experiment shows a more gradual dependence $\Delta T_{S}\left(\phi \right)$. Both the experiment and theory show that the effect of fillers on the spinodal vanishes when ϕ∼0.6.

## 4. Conclusions

The theory developed in the present work calculates the effect of fillers on the spinodal transition temperature of a compressible polymer blend. This calculation relies on taking into account the osmotic effect of fillers on the thermodynamic state of the filled compressible polymer blends described in Section 2.2. Based on the developed theory, the spinodal of a filled polymer blend, which is given by Equations (10) and (11), was derived. This spinodal has been used to explain the experimental observation in [3] that the presence of fillers increases the transition temperature of a blend, thus enhancing its stability.

The main advantage of the present theory is that it analytically describes the effect of fillers on the stability of a *compressible* polymer blend. Taking into account the compressibility of a blend is known [28] to be a necessary condition for making adequate predictions of the low critical solution temperature (LCST) phase behavior of this blend. The above LCST behavior is often observed in experiments such as, in particular, the experiment in [3], analyzed in the present work. In addition, the present theory is based on a rigorous thermodynamic derivation of the correction to the spinodal stability condition of a compressible polymer blend, caused by the presence of fillers. This advantage proves possible to adequately predict the experimentally observed behavior of the spinodal transition temperature as a function of the fractions $\phi_{1,2}$ of polymer species in a blend.

According to our results, solid fillers can be effectively used to improve the miscibility of polymer blends that show LCST behavior. This statement is based upon one of the central results of the present work, i.e., the prediction of the spinodal temperature $T_{S}\left(\phi_{1}\right)$ of a filled blend. This result is illustrated in Figure 2. According to this figure, the presence of fillers increases the spinodal transition temperature in the whole range of the volume fractions of polymer species. $T_{S}\left(\phi_{1}\right)$ is found to be a non-monotonic function of $\phi_{1}$ that features a minimum. Moreover, the predicted shift of $T_{S}$, caused by the presence of fillers, shows the same trend as that observed in the experiment in [3]. In particular, both the experiment and the theory show a similar non-monotonic dependence of $T_{S}\left(\phi_{1}\right)$, as well as vanishing the effect of fillers at $\phi_{1}$ ∼ 0.6. The described good agreement between the theory and experiment illustrated in Figure 2 proves the predictive power of the developed approach.

The theoretical method developed in the present work opens a route toward a comprehensive understanding of the effect of fillers on the stability and miscibility of realistic compressible polymer blends. The present version of the developed approach focuses on the simplest kind of interactions between fillers and polymers, i.e., the excluded volume (osmotic) interactions. We show that the presence of these interactions, overlooked in previous works, alone can explain the experimental observations of the spinodal of a filled polymer blend. These interactions could, therefore, be even more important than the enthalpic (e.g., adsorption) and entropic (e.g., depletion) interactions. Still, the developed method as such is not restricted to only the excluded volume interactions between the fillers and polymers considered in the present work. Moreover, we surmise that sufficiently strong enthalpic interactions between fillers and polymers can significantly add to the variety of effects of fillers on the miscibility and stability of polymer blends. Extending the developed approach by taking into account the enthalpic and entropic surface interactions between fillers and polymers presents a clear perspective of further development based on the reported work.

## Data Availability

Data are contained within the article.

## Abbreviations

The following abbreviations are used in this manuscript:

SL	Sanchez–Lacombe lattice theory
LCST	Low critical solution temperature
UCST	Upper critical solution temperature
r.(l.) h.s.	Right (left) hand side

## Appendix A

In this Appendix, we derive the expression for the spinodal condition given by Equation (2). By directly changing the pair of variables $\left(P,M_{i}\right)\to \left(\eta ,\phi_{i}\right)$ (i=1,2), while keeping the other variables unchanged, one finds the relation

(A1) $$M_{i}\mu_{ii}=\phi_{1}\phi_{2}\left(\partial_{\phi_{i}}\mu_{i}-{\left(\partial_{\eta }\tilde{P}\right)}^{-1}\partial_{\phi_{i}}\tilde{P}\partial_{\eta }\mu_{i}\right).$$

From Equation (A1) one finds

(A2) $$\sum_{i=1,2}r_{i}^{-1}M_{i}\mu_{ii}=\phi_{1}\phi_{2}\left(\sum_{i=1,2}r_{i}^{-1}\partial_{\phi_{i}}\mu_{i}-{\left(\partial_{\eta }\tilde{P}\right)}^{-1}\partial_{\phi }\tilde{P}\partial_{\eta }\left(r_{1}^{-1}\mu_{1}-r_{2}^{-1}\mu_{2}\right)\right).$$

Using the Gibbs–Duhem relation written in the form

(A3) $$Td\tilde{P}=\eta \sum_{i=1,2}r_{i}^{-1}\phi_{i}d\mu_{i},$$

one finally arrives at

(A4) $$M_{1}\mu_{11}=r_{1}\phi_{1}\phi_{2}^{2}\left(\partial_{\phi }\left(r_{1}^{-1}\mu_{1}-r_{2}^{-1}\mu_{2}\right)-T\eta^{-1}{\tilde{\kappa }}_{T}{\left(\partial_{\phi }\tilde{P}\right)}^{2}\right).$$

The obtained Equation (A4) proves that the inequality given by Equation (2) is identical to the spinodal condition $\mu_{11}\geq 0$.
